# Realization of Lodging Tolerance in the Aromatic Grass, *Cymbopogon khasianus* Through Ploidy Intervention

**DOI:** 10.3389/fpls.2022.908659

**Published:** 2022-05-09

**Authors:** Yerramilli Vimala, Umesh Chandra Lavania, Madhavi Singh, Seshu Lavania, Sarita Srivastava, Surochita Basu

**Affiliations:** ^1^Department of Botany, Chaudhary Charan Singh (CCS) University, Meerut, India; ^2^Department of Botany, University of Lucknow, Lucknow, India; ^3^Department of Botany, Chowdhary Mahadev Prasad (CMP) College (Constituent College of Central University of Allahabad), Prayagraj, India; ^4^Department of Botany, Tripura University (A Central University), Agartala, India

**Keywords:** polyploidy and lodging tolerance, ploidy mediated histological changes, polyploidy and secondary metabolites, polyploidy breeding, aromatic grass

## Abstract

Artificial polyploidy that brings about increase in cell size confers changes in histo-morphology leading to altered phenotype, causing changes in physiological attributes and enhanced concentration of secondary metabolites. The altered phenotype is generally a manifestation of tissue hardiness reflected as robust plant type. Based on a case study undertaken on an industrially important grass, *Cymbopogon khasianus* (2*n* = 60) valued for its citral rich essential oil, here we report that the artificial polyploidy not only brings about enhancement in concentration of essential oil but also facilitates lodging tolerance. The latter is contributed by ploidy mediated changes that occur to the cells and tissues in various plant organs by way of increased wall thickening, tissue enhancement and epidermal depositions that enable robust features. An exhaustive illustrated account covering various micro-/macro-morphological, skeletal and histochemical features constituting growth and development vis-a-vis ploidy mediated changes is presented highlighting the novelties realized on account of induced polyploidy.

## Introduction

It is an established fact that multiple cyclic episodes of whole-genome doubling or polyploidization have led to evolution and speciation in flowering plants (Wendel, 2015). The wondrous cycles of genome doubling are thought to be correlated with periods of extinction or global climate change over the geological time scale, while polyploids often thrive in harsh or disturbed environments (Van de Peer et al., 2021), and could colonize new habitats (Moraes et al., 2022). There are contrasting viewpoints about polyploidy as an evolutionary force, spanning from “evolutionary dead end” to “major player in evolution” (Lavania, 2020), but an established ecological force (Van de Peer et al., 2021). Initial models of polyploid evolution based on studies spanned over 70 years, considered autopolyploidy as an evolutionary dead end (Stebbins, 1999), but subsequent studies led to believe that polyploidy *per se* both auto- and allo- are the source for evolutionary innovation and species diversification (Van de Peer et al., 2021). Evidence accumulated on ecological niche vis-a-vis polyploid establishment underpin that polyploids evince broader adaptability and vast ecological tolerance (teBeest et al., 2012) and owe higher invasive potential compared to their diploid relatives (Pandit et al., 2011), although the distribution of such polyploid lineages may have restricted range distribution in preferred environment (Villa et al., 2022). It is also surmised that there is differential response to both biotic interactions and abiotic stress on polyploids vs. non-polyploids (Van de Peer et al., 2021). Polyploid organisms are thought to be more resilient to extreme environments owing to increased genetic variation, bufferring effect and adaptive potential of duplicated genes (Van de Peer et al., 2017; Doyle and Coate, 2019). As such, stress response in general is an important factor in the establishment of polyploidy (Van de Peer et al., 2021).

Identification of several critical genome replication/duplication events during the periods of major environmental and climate change (Alix et al., 2017), have led to propose that environmental constraints could elicit polyploidization/genome duplication as an escape to overcome the vagaries of harsh environment as adaptive speciation and survival strategy (Levin, 2019).

While discussing the likely patterns of speciation in next 500 years, Levin (2019) opines that if the global climate undergoes major changes, then this will lead to an increase in the number of plant chromosomes, and thereby an increase in the current proportion of polyploids in angiosperms to 35–50%, and an overall proportion up to 50% of the Earth’s plant species as polyploids. He further argues that such polyploidy incidences would be more pronounced in short-statured herbaceous plants. This is consistent with our earlier observations (Lavania and Srivastava, 1988, 1990), where it is observed that the subcultures of the diploid vs. autoteraploid calli when grown over a passage of monthly subcultures under stressful environment *in vitro*, exhibit differential effect to polyploidization, whereby the diploids turn into polyploids but the tetraploids retain the original ploidy status.

The polyploidy/genome doubling is considered a natural consequence to overcome abiotic stress. It is known to bring about changes in transpiration, water use efficiency, photosynthetic rate, phenology, antioxidant response, and morphology etc. that confer success to polyploids (Maherali et al., 2009; Deng et al., 2012; Soltis and Soltis, 2014). A lot has been discussed about the significance of both auto- and allo- polyploidy in conferring novelty and value addition to plants (Levin, 1983, 2002; Lavania and Vimala, 2022), especially where the plant biomass is the source of economic product, and their active metabolite components are valued in industrial applications (Lavania, 2005). It was therefore planned to explore whether polyploidy could lead to changes that confer morphological robustness from cultivation perspective, targeting a species that is cultivated through vegetative tillers under commercial cultivation, and the sexual system is deficient.

The genus *Cymbopogon* Spengel comprises a group of aromatic grasses that are either densely or loosely tufted. Although, almost all the species produce essential oil in the secretory cells present in the vegetative tissues of shoot, leaf and inflorescence, but only six species are majorly used for commercial cultivation, and the *C. khasianus* is one of them (Kumar et al., 2000; Lavania et al., 2012; Yogendra et al., 2021). Soenarko (1977) has provided detailed information on morphological, anatomical, geographical and ecological data on 55 species from taxonomic perspective, pinpointing that most of the species are perennial, where lateral branches/tillers are appressed to the main axis (culm). The perennial growth habit makes the growing tiller prone to lodging, and more so in *C. khasianus* because it becomes taller than the other species in the fast-growing season during warm and humid conditions.

The present study was undertaken to explore ploidy mediated approach to help realize genetic enhancement of an elite clone of an industrially important aromatic grass that suffers from lodging, and examine histo-morphologocal, qualitative and yield contributing characters from breeding perspective.

## Materials and Methods

### Plant Material

An elite clone namely “CIM-Suwarna” of *Cymbopogon khasianus* (Hack) Stapf (ex Bor), (2*n* = 60), developed at the CSIR-Central Institute of Medicinal and Aromatic Plants, Lucknow, India (Lal et al., 2010) was targeted to develop its clonal autotetraploids. Another clone “Krishna” of a related species *Cymbopogon flexuosus* said to produce highest concentration of lemongrass essential oil and popular in cultivation was used as a “Check.”

### Realization of Clonal Polyploids

The target species is an aromatic grass that sports laterally proliferating tiller formation, where meristem is basal and lay deep seated beneath the leaf sheath. As such special efforts are required for colchicine administration for induction of polyploidy. Accordingly, the clonal polyploids were developed following the experimental protocol standardized by Lavania et al. (2012). The axillary buds on fast-growing slips (tillers) were exposed to target the basal meristem, followed by immersion of bud bearing region of such slips in 0.1% (v/w) aqueous solution of colchicine in 2% DMSO for 7 h at 25°C, followed by thorough washing in running water and planting in soil. Emerging plantlets were screened for leaf stomata size, and those with uniformly and distinctly enlarged stomata, roughly twice the volume of source diploids, were selected followed by cytological screening to isolate polyploids. Both diploid and polyploid (auto-tetraploid) clones derived from the “same source tiller” were screened out through six passages of clonal propagation spread over 2 years for ploidy stability, and planted in field for further observations.

### Micromorphological and Productivity Analysis

Cytologically stable autopolyploids vis-a-vis source diploids were scored for ploidy associated changes in morpho-anatomical features associated with plant biomass, cell geometry of vascular and non-vascular tissue, and essential oil secretory cells by light and fluorescence microscopy.

*(i) Tissue preparation for histological examination for essential oil secretory cells:* Hand cut sections of leaf and culm were prepared and incubated for 30 min at room temperature in 0.75% Schiff’s reagent. The sections were then washed three times (10 min) with a freshly prepared solution of 0.5% (w/v) sodium metabisulfite in 0.1% HCl and mounted in 1N.HCl according to Lewinsohn et al. (1998). The stained sections were examined under microscope using both transmitted light and epifluorescence (blue or UV excitation). The size and frequency of essential oil glands was recorded at 40X magnification. Size of the essential oil secretory cells was calculated with the help of ocular micrometer, and cell frequency estimated by counting the number of cells/cm^2^ of the leaf area. Following similar staining procedures, the essential oil cells could also be seen in the surface view.

*(ii) Tissue preparation for epicuticular depositions, cell geometry*: To examine phytoliths on leaf surface, cleaned leaf pieces were boiled for 1–2 h by gradually raising the temperature of the waterbath from 80 to 100^°^C to digest the soft tissues according to Parry and Smithson (1958). Thereafter the leaf pieces were carefully washed and stained in 1% safranin in 70% glycerol, and observed under microscope for recording quantitative measurements. For recording data on cell geometry fresh leaves were scratched to clear the adaxial epidermal surface to record observations on abaxial surface.

To scan epicuticular wax deposition on leaf, the third mature leaf from a well grown tiller was excised, washed thoroughly in dH_2_O distilled water, rinse dried and fixed in 5% Glutaraldehyde overnight and then transferred to phosphate buffer (pH 7.5), followed by dehydration through alcohol series. Air dried samples cut into 2–3 mm^2^ pieces were loaded on SEM Aluminum stubs using double sided adhesive tape. Samples were coated in POLARON SC-7640 Sputter coater at 18 mA current for 160 s in which Gold-Palladium alloy was used as coating material. The samples were then scanned using a conventional scanning electron microscope LEO-430. The leaf abaxial surface ultra-structural details were examined and exposures were captured at desired magnifications.

To record the width of leaf mid-vein, middle region of the leaf was selected (Wilkinson, 1979). The glycerine mounts of hand-cut vertical sections (V.S.) stained in 1% safranin were examined under Nikon Eclipse Ni (Japan) microscope for anatomical characters. The measurements were recorded with a calibrated eyepiece at 100×.

*(iii) Biomass and essential oil yield*: For recording observations on biomass and essential oil yield, both progenitor diploid and its corresponding autopolyploid clones, along with a standard “Check” (clone Krishna of *C. flexuosus*) were grown in 10-m^2^ plot with 36 hills per plot and plant to row distance 50 cm at the experimental field of the CSIR-Central Institute of Medicinal and Aromatic Plants, Lucknow, India. Data on herbage yield were taken at 4-monthly harvests over 1 year. Essential oil concentration in the leaves harvested at a similar growth stage was estimated by hydrodistillation in Clevenger’s equipment adjusted to 60°C and run for 2 h, and qualitative analysis of essential oil was done by GLC.

## Results

**FIGURE 1:**
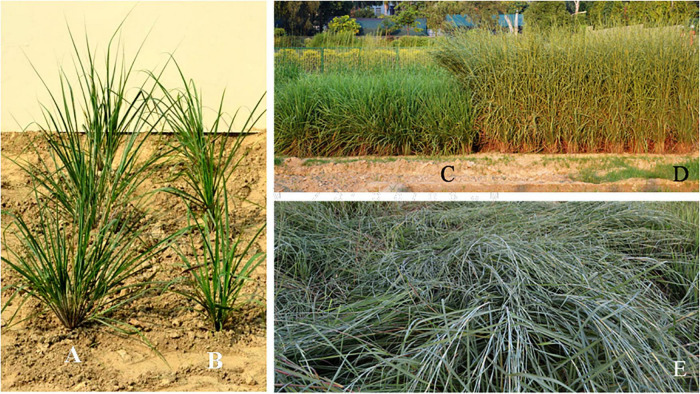
Development of lodging tolerant autotetraploid *Cymbopogon khasianus.* **(A)** One month old plant of the tetraploid (4*n* = 120) and **(B)** corresponding source diploid (2*n* = 60). **(C–E)** Field view of fully grown plants, **(C)** check clone “Krishna”, **(D)** lodging tolerant autotetraploid, and **(E)** lodging diploid progenitor.

**FIGURE 2:**
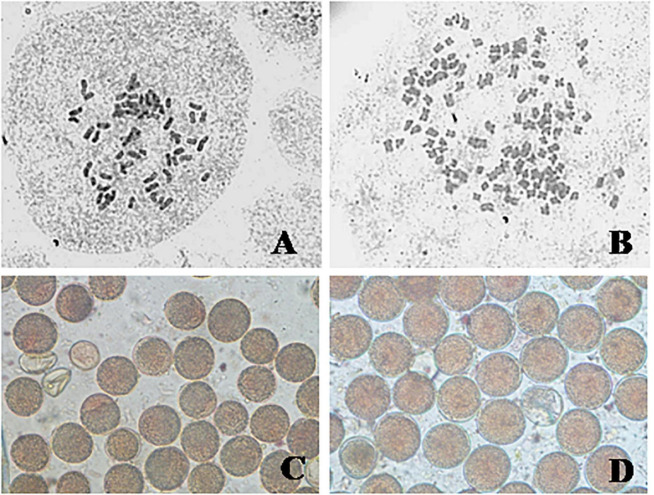
Somatic chromosomes and pollen grains of *C. khasianus:* somatic chromosomes **(A)** source diploid (2*n* = 60) and **(B)** autotetraploid (4*n* = 120); pollen grains – **(C)** diploid, **(D)** tetraploid.

**FIGURE 3:**
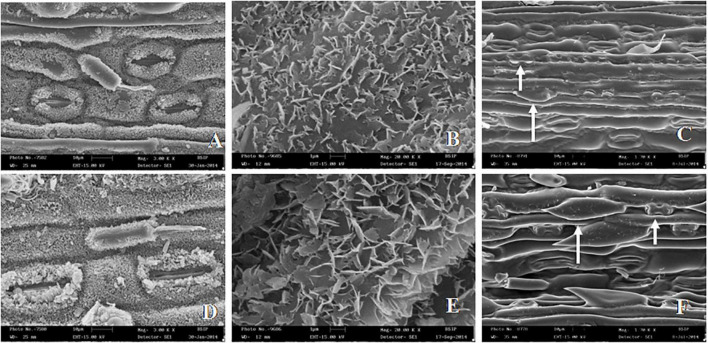
SEM images of leaf surface features in *Cymbopogon khasianus* in the diploid (upper column) vs. autotetraploid (lower column) showing: **(A–D)**. Stomata with cuticuler wax deposition, **(B–E)**. Wax crystals, and **(C–F)**. Phytolith (small arrow) and macro-hair (large arrow).

**FIGURE 4:**
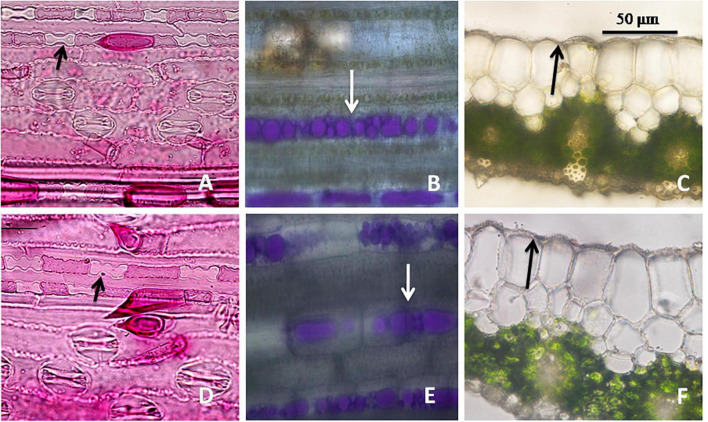
Leaf epidermal features in *Cymbopogon khasianus* in the diploid (upper column) vs. autotetraploid (lower column) showing: **(A–D)**. Phytolith (arrow marked)/stomata, **(B–E)** secretory channels filled with essential oil (stained magenta), **(C–F)** cuticle thickness.

**FIGURE 5:**
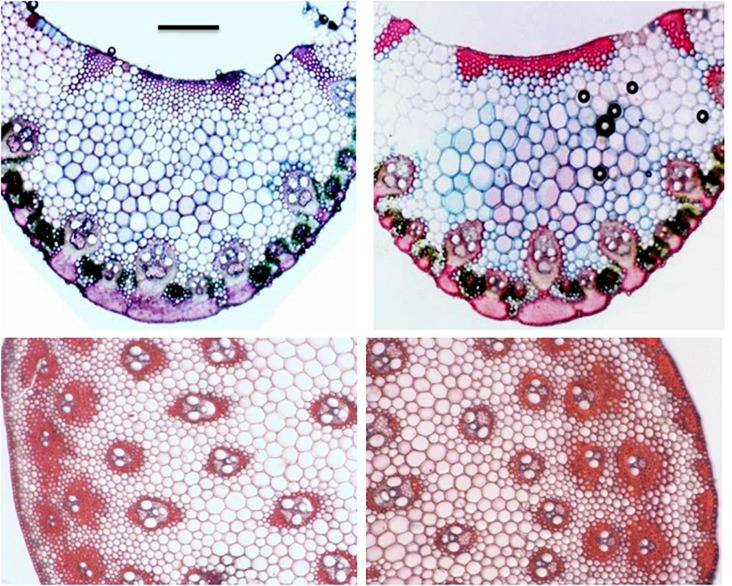
VS of leaf (mid rib section) and TS of culm (partial) in the diploid (left) and tetraploid (right). Note, enhanced thickening/sclerenchymatus regions in the hypodermal and vascular bundle region in the tetraploid. Sclae bar = 200 μ.

Both the diploid and its corresponding autopolyploid grown under similar conditions were examined for recording the data on morphological, histological and metric traits and productivity analysis. A general account of observations recorded for morphologial and histological features is provided in Table 1, and the key features related to realization of sturdiness are given in Table 2. The data related to yield contributing characters and breeding potential of the developed polyploid clone are provided in Table 3. All the qualitative features related to the theme of the study are depicted in Figures 1–5.

**TABLE 1:** Exomorphology, anatomy and growth related patterns affected by ploidy change (± *SE*) in the *Cymopogon khasianus*.

S. No.	Characters	Diploid	Tetraploid
1	Color (as per RHS catalog: Leaf sheath	Yellow green 146C	Yellow green 146C
2	Leaf (adaxial)	Green group N137B	Green group N137B
3	Leaf (abaxial)	Green group 137A	Green group 137A
4	Stem color	Yellow green 146D	Yellow green 146D
5	Spikelet color	Grayed green	Grayed green
6	Flowering time	Nov-Dec	September
7	Number of tillers (1 year)	97 ± 0.44	81 ± 0.65
8	Number of leaves per tiller	3–7	4–8
9	Plant height (cm)	147 ± 0.44	158 ± 0.71*
10	Culm length (cm)	228 ± 0.53	255 ± 0.63*
11	Inflorescence Length (cm)	75 ± 0.30	97 ± 0.53*
12	Number of nodes in culm	15 ± 0.41	12 ± 0.31
13	Length of internode in the middle region of culm (cm)	26.0 ± 0.48	29.5 ± 0.58*
14	Average Diameter (cm) of culm between 2nd and 3rd node	0.44 ± 0.009	0.49 ± 0.004*
15	Number of vascular bundles in the culm	115 ± 0.29	145 ± 0.88*
16	Area of the culm cross section occupied by the vascular bundles (mm^2^)	6.54 ± 0.12	6.35 ± 0.09*
17	Area (L X B) of single culm vascular bundle of the 3rd concentric ring (μm^2^)	22,500 ± 687	31,500 ± 814*
18	Area of Meta-xylem vessel (L × B) culm Vascular Bundle of the 3rd concentric ring (μm^2^)	1,524 ± 54	1,857 ± 24*
19	Leaf mesophyll thickness/vascular thickness (μm)	165 ± 6.03/90 ± 0.0	195 ± 5.48*/115 ± 1.38*
20	Average thickness of leaf cuticle: adaxial/abaxial (μm)	3.7 ± 00/1.85 ± 0.01	5.55 ± 0.02*/3.7 ± 00*
21	Root Stele diameter (mm)	0.936 ± 0.038	1.051 ± 0.014*
22	Thickness of root vascular tissue (mm)	0.202 ± 0.005	0.244 ± 0.003*
23	Number of vascular bundles in root	18.8 ± 0.38	16.3 ± 0.21
24	Percentage of leaf vascular tissue	54.54%	58.97%
25	Average Leaf length × width (cm)	100 ± 2.0 × 1.24 ± 0.07	92 ± 1.8 × 1.31 ± 0.3
26	Average number of leaf major vein	16 ± 0.21	13 ± 0.21
27	Average distance between major veins (mm)	2.042 ± 0.026	2.210 ± 0.044*
28	Average leaf area (cm^2^)	87.18 ± 0.31	49.38 ± 0.29*
29	Lumen size of essential oil containing cell (μm^2^)	947 ± 21	1,747 ± 32*
30	Essential oil concentration in fresh herb (%)	0.52 ± 0.03 Citral = 82.0% Geraniol = 3.9%	0.66 ± 0.05* Citral = 85.9% Geraniol = 2.7%
31	Area of Leaf midrib in vertical section (μm^2^)	742,500 ± 3,163	1,026,000 ± 1,391*
32	Average number of oil cells in leaf sheath	205 ± 0.683	178.7 ± 1.21
33	Area occupied by bulliform cell/cm^2^ of leaf vertical section	0.3145 ± 0.016	0.3093 ± 0.006*
34	Area of stomatal complex (μm^2^)	804.96 ± 27.9	1260.84 ± 34.8*
35	Stomatal index	27.65 ± 1.29	24.9 ± 0.802*
36	Stomatal guard cell area (μm^2^)	174.1 ± 12.2	254.8 ± 35.8*
37	Size of leaf epidermal cell (μm^2^)	1,911 ± 119.7	2,249 ± 90.8*
38	Phytolith size (μm^2^) on leaf abaxial surface	261.07 ± 22.74	374.11 ± 21.17*
39	Phytolith frequency/mm^2^ of leaf abaxial surface	106 ± 9.24	80 ± 13.3
40	Macrohair frequency/mm^2^ of leaf abaxial surface	45.61 ± 1.49	31.45 ± 3.86
41	Size of macrohair (μm^2^)	530.22 ± 24.73	850.97 ± 34.45*
42	Wax frequency/10μm^2^ (on epidermal surface)	10.42 ± 0.11	7.63 ± 0.32

*Values significantly different with respect to diploid by Student’s t-test at P = 0.05.

**TABLE 2:** Histomorphological features in the progenitor diploid and corresponding autotetraploid related to plant hardiness enabling lodging tolerance in the tetraploids.

Name of the species	Ploidy status	Hypodermal sclerenchyma thickness of culm cross section (μm) ± *SE*	Thickness of vascular region in culm cross section (mm) ± *SE*	Thickness of hypodermal sclerenchyma of leaf adaxial/abaxial (μm) ± *SE*	Average wax flake length (μm) on stomata/ epidermal surface ± *SE*	Area occupied by phytoliths (mm^2^)/cm^2^ of leaf abaxial surface ± *SE*	Increase in thickness of hypodermal sclerenchyma in culm in 4*n* over 2*n* (%)	Increase in thickness of culm vascular region (%)	Increase in hypodermal sclerenchyma of leaf in 4*n* over 2*n* (%)	Increase in wax flake length in 4*n* over 2*n* (%)	Increase in the area occupied by phytoliths in leaf in 4*n* over 2*n* (%)
*C. khasianus*	2*n*	25.9 ± 0.78	1.725 ± 0.094	89.02 ± 0.92 /69.44 ± 1.17	1.45 ± 0.095 /0.921 ± 0.062	2.82 ± 0.113	54.28	17.39	54.60	103.71	33.33
	4*n*	39.96 ± 0.80*	2.025 ± 0.227*	117.6 ± 1.49* /120.3 ± 0.70*	3.37 ± 0.095* /1.46 ± 0.095*	3.76 ± 0.140*					

*Values significantly different with respect to diploid by Student’s t-test at P = 0.05.

**TABLE 3:** Essential oil secretory channels and essential oil/biomass yield in the progenitor diploid and corresponding autotetraploid in *Cymbopogon khasianus.*

Name of the species	Ploidy status	Lumen size of essential oil-containing cell (μm^2^) ± *SE*	Frequency of essential oil channels/cm^2^ of leaf VS ± *SE*	Essential oil concentration in fresh herb (%) ± *SE*	Area (cm^2^) covered by essential oil-producing cells/cm^2^ of leaf VS ± *SE*	Number of tillers arising from a slip in 60 days ± *SE*	Fresh herb biomass (kg 10 m^–2^) ± SE	Increase in essential oil channel area under cover in 4*n* over 2*n* (%)	Increase in essential oil concentration in 4*n* over 2*n* (%)	Increase in biomass yield in 4*n* over 2*n* (%)	Increase in essential oil productivity in 4*n* over 2*n* (%)
*C. khasianus*	2*n*	947 ± 21	3,168 ± 97	0.52 ± 0.03	0.030 ± 0.0002	13.8 ± 0.13	38.96 ± 0.23	26.67	26.92	20.30	33.99
	4*n*	1,747 ± 32*	2,175 ± 21*	0.66 ± 0.05*	0.038 ± 0.0004*	26.4 ± 0.22*	46.87 ± 0.34				
*C. flexuosus—*clone *Krishna*	2*n*	412 ± 17	4,419 ± 12	0.65 ± 0.02	0.018 ± 0.0001	12.3 ± 0.15	20.00 ± 0.25				

*Values significantly different with respect to diploid by Student’s t-test at P = 0.05.

It is observed that there is an overall increase in body size of the developed polyploid. This is consummated through increased cell size at all the organizational levels, including tissues, organs, and micromorphological constituents, and their associated developmental changes in cell wall thickening, and metabolic changes related to photosynthetic efficiency and secretion of secondary metabolites in the form of the essential oil. Whereas, tissue thickening enables plant sturdiness, an increased cell size adds to biomass and physiological efficiency for enhanced essential oil concentration, leading to overall lodging tolerance and enhanced productivity of the economic product. Specific details based on these observations are dealt in discussion section.

## Discussion

Vast data accumulated on the biology of polyploids for over more than 100 years suggest that polyploids are sturdier than diploids owing to the thicker stem and leaves and associated corresponding changes (Ramsey and Ramsey, 2014), sporting enhanced efficiency in stressed environment (Pandit et al., 2011; teBeest et al., 2012; Levin, 2019), as also offering the advantage of evolutionary novelty (Levin, 2002), and profound amenability in diverse habitats, albeit having restricted distribution (Villa et al., 2022). It is with this background that the present study was undertaken on a supposedly paleo-hexaploid (2*n* = 60) species of the *Cymbopogon* species complex (base number × = 10, Soenarko, 1977; Lavania, 1988) to further enhance its biological potential by ploidy elevation for wider adaptability and enhanced productivity.

The results presented here demonstrate successful realization of genetically stable clonal autopolyploids in a commercially important aromatic grass valued for its citral rich essential oil used for its multifarious applications in aroma industry. Whereas, the target species used in this study is known to produce high biomass compared to other species of the genus, but the high biomass producing canopy *per se* is fraught with danger of lodging when challenged by intense air velocity and rains encountered in the fast-growing season. The instant induced polyploid reported here has been found to overcome such lodging challenge on account of stout stem, thicker and broader leaves realized in the polyploid owing to enhanced sclerenchyma in the vascular and epidermal regions and waxy coating/siliceous phytolith in the cuticular surface. At the same time the induced polyploid offers commercial advantage of enhanced biomass and increased concentration of essential oil, thus a dual advantage from cultivation perspective.

Comparative assay of micromorphological characters in diploids and autotetraploids reveal a clear-cut increase in the cell size reflected at all levels, including epidermal cells, vascular tissue, stomatal guard cells, essential oil secretory cells, phytolith size, cuticle thickness, and even epicuticular waxy deposition, albeit there is decrease in frequency of all such cells in per unit area. Nevertheless, there is an overall enhancement in the area occupied by the cells constituting larger tissues and organs. In particular, for the tissues contributing to economically important part, i.e., essential oil secreting cells in the source biomass, there is an overall increase in the area occupied by the essential oil secreting cells by 26.7%, and biomass yield by 20.3%, essential oil concentration by 26.9%, enabling an overall increase in essential oil productivity by 34%. At the same time the instant polyploid clone could withstand lodging pressure challenged by strong air currents and heavy rains encountered during the growing season (Figure 1D). Further, when compared with an elite clone “Krishna” of a related species *Cymbopogon flexuosus*—taken as “Check” (Figure 1C), the polyploid clone of *C. khasaianus* is superior by 237% in terms of the productivity of the economic product, i.e., citral rich essential oil.

The exhaustive data presented amply suggest that there is an overall increase in body size of the developed polyploid consummated through increased cell size at organizational levels, including tissues, organs and micromorphological constituents, and their associated developmental and metabolic changes. The major constituent components that deserve particular attention are highlighted below:-

### Vascular Thickening Adds to Robustness and Lodging Tolerance

The findings presented in this study provide an exhaustive account of cell size associated comparisons between the diploid and the derived isogenic clonal polyploid (Tables 1, 2). Such an elaborated account on ploidy associated changes has not been presented before. It is observed that with the increase in cell size in the tetraploid there are associated changes in the constituting tissues and organs, including cell wall thickening, secretions and waxy/siliceous phytolith depositions, and more particularly the hypodermal sclerenchyma thickness in the culm and leaf by a factor of > 50% (Table 2). It would be obvious that such enhanced thickening in the vascular tissues and depositions in the cuticular regions would impart physical robustness. The relative ratio toward thick walled sclerenchymatous regions would further add to robusticity, and in turn tolerance to physical pressures. Such a manifestation is in tune with the ecological behavior of neopolyploids known to demonstrate larger phenotypic and ecological ranges (Barker et al., 2012), through avoidance of competition with their established diploid parents (Hegarty et al., 2008).

The extracellular matrix constituting the cell wall is mainly composed of polysaccharides and structural proteins (Burton et al., 2010). The cell types appear to have a typical size range closely associated with function (Amodeo and Skotheim, 2016), characteristically optimized for cell fitness (Miettinen and Björklund, 2016; Vargas-Garcia et al., 2018). This is achieved through constant adjustment of cell wall composition and rearrangement of wall polysaccharides (Cosgrove, 2018; Zhang et al., 2021). Baker et al. (2017) have hypothesized that tetraploidy results in trait disintegration allowing for transgressive phenotypes emanating from changs in morphological, anatomical and physiological traits. A balanced ratio of structural components in the cell wall rearrangement facilitates wall rigidity, flexibility for cell dynamics, and enhanced potential for growth and development (Westermann, 2021), that could enable wider adaptability and robusticity to the polyploids. All this fits well in present set of things.

### Cell Size Contributes to Enhanced Physiological Efficiency and Increased Productivity

The key observation made out in this study is that there is an increase in cell size/wall thickening of the constituent cells of different tissues and organs on account of ploidy change. Such an effect is global and is observable in the overall organization of the polyploid clone (Tables 1, 2). This is more clearly discerned in the size of stomatal guard cells, epidermal cells and essential oil secretory cells, and physiological activity in terms of chlorophyll content and waxy secretion/phytolith deposition. The increase in lumen size of the secretory cell is approximately double the volume compared to the source diploids, but in terms of overall area occupied by such cells this is accounted to ∼25%, which is also reflected in terms of essential oil concentration and yield of biomass and productivity of the economic product (Table 3).

A clear instance of increase in cell size as measured at the level of stomatal guard cells with ploidy change across the species has earlier been observed in *Coffea* spp. (Mishra, 1997), and in relation to essential concentration in *Cymbopogon* spp. (Janaki-Ammal and Gupta, 1966), and at autopolyploid level in different species of *Cympopogon* (Lavania et al., 2012). Extensive study undertaken by Soenarko (1977) on systematics and Lavania (1988) on cytology, lists the occurrence of natural ploidy series across the species with 2*n* = 20, 40, 60, wherein *C. khasianus* belongs to 2*n* = 60 series. A comparison of stomatal guard cells and essential oil secretory cells shows corresponding increase in cell size with ploidy series. Obviously, the stomata/essential oil secretory cells in *C. khasianus* are distinctly larger compared to natural diploids, and the same is reflected in the derived autopolyploids sporting further enlargement (Lavania et al., 2012), and is commensurate to ploidy elevation.

Doyle and Coate (2019) provide an elaborated account on the impact of genome doubling on the biology of cell vis-a-vis physiological and morphological novely. Trojak-Goluch et al. (2021) have discussed the prospective applications of artificial polyploidy in plant breeding of industrial crops. Whereas, manifestation of “gigas” effect in the phenotype is a common feature reflected in the neopolyploids but decrease in the ratio of nuclear membrane to chromatin brings more surface area to come in contact to genetic activity enabling higher physiological activity and developmental changes (Lavania et al., 2012; Doyle and Coate, 2019) in general, and secondary plant products in particular (Lavania, 2005). Since stomata are associated with various physiological activities in cell like water and CO_2_ exchange, therefore, ploidy level may influence physiological activities too (Lea et al., 1977). Of course, manifestation of such effect could be genotype and species dependent (Lavania, 2005; Lavania et al., 2012).

## Concluding Remarks

Artificial polyploidy is known to increase cell size in plants; axiomatically it is likely to enhance cell size of organs and tissues involved in metabolite production. However, effect of such polyploidy mediated changes could be species specific that could be inferred from micro-morphological examination of key components. *Cymbopogon* is a perennial C4 aromatic grass with numerous stiff stems arising from a short, rhizomatous root stock, indigenous to tropical and sub-tropical parts of the world. In these aromatic grasses the essential oil is stored in secretory channels that run parallel to the leaf mesophyll on the abaxial side embedded in between the vascular cylinders. The essential oil synthesis is at its maximum secretion with the onset of blooming. However, the fast growing tillers reaching the blooming stage are prone to lodging when challenged by strong winds and heavy showers. Therefore, realizing plant hardiness is considered an important requirement for optimum harvest. The present study on the development of lodging tolerant plants is an important step that could be achieved through induced polyploidy, vis-a-vis adding to enhanced productivity without causing any adverse effect (related to seed-based cultivation posed on account of autopolyploidy) on cultivation since the cultivation of this grass is done through vegetative plantation as the standard practice.

## Data Availability Statement

The raw data supporting the conclusions of this article will be made available by the authors, without undue reservation.

## Author Contributions

YV conceptualized and reviewed the manuscript. UL conceived the experimentation, performed the field evaluation, and drafted the manuscript. MS and SL performed micromorphological analysis and histological studies. SS and SB jointly did cytological screening. All authors contributed to the article and approved the submitted version.

## Conflict of Interest

The authors declare that the research was conducted in the absence of any commercial or financial relationships that could be construed as a potential conflict of interest.

## Publisher’s Note

All claims expressed in this article are solely those of the authors and do not necessarily represent those of their affiliated organizations, or those of the publisher, the editors and the reviewers. Any product that may be evaluated in this article, or claim that may be made by its manufacturer, is not guaranteed or endorsed by the publisher.
